# Supplementary material for: Role of optimization algorithms based fuzzy controller in achieving induction motor performance enhancement
Source: Nat Commun. 2020 Jul 30;11:3792. doi: 10.1038/s41467-020-17623-5 (PMC7393368; doi:10.1038/s41467-020-17623-5)
Supplement: Supplementary file 1 — Supplementary Information [file 41467_2020_17623_MOESM1_ESM.docx]

**Supplementary Information**

**Role of Optimization Algorithms based Fuzzy Controller in Achieving Induction Motor Performance Enhancement**

**Hannan et al.**

**Supplementary Figures 1 to 15**

**Supplementary Tables 1 to 11**

**Supplementary Notes 1 to 5**

**Supplementary References**

**Supplementary Figure 1.** Flow diagram of the proposed QLSA algorithm based optimum fuzzy speed controller design procedure

**Supplementary Figure 2.** Flow diagram of LSA-based optimum fuzzy speed controller design procedure

**Supplementary Figure 3.** Flow diagram of BSA-based optimum fuzzy speed controller design procedure

**Supplementary Figure 4.** Flow diagram of GSA-based optimum fuzzy speed controller design procedure

**Supplementary Figure 5.** Flow diagram of PSO-based optimum fuzzy speed controller design procedure

|  |  |  |  |
| --- | --- | --- | --- |
| (a) | (b) | (c) | (d) |

**Supplementary Figure 6.** **The global optimization performance assessment of QLSA, LSA, BSA, GSA and PSO under different benchmark functions.** **a.** The global optimisation results for QLSA, LSA, BSA, GSA and PSO in benchmark function F11 (Ackley) is obtained based on dimension problem, search space and function minimum (Supplementary Table 1). **b.** The global optimisation results for QLSA, LSA, BSA, GSA and PSO in benchmark function F12 (Griewank). **C.** The global optimisation results for QLSA, LSA, BSA, GSA and PSO in benchmark function F13 (Penalised) and. **d**. The global optimisation results for QLSA, LSA, BSA, GSA and PSO in benchmark function F14 (Penalised 2)

|  |  |  |  |
| --- | --- | --- | --- |
| (a) | (b) | (c) | (d) |

**Supplementary Figure 7.** **The convergence characteristics performance evaluation of QLSA, LSA, BSA, GSA and PSO under different benchmark functions. a.** Convergence characteristic curves for QLSA, LSA, BSA, GSA and PSO in benchmark function F11 (Ackley). **b.** Convergence characteristic curves for QLSA, LSA, BSA, GSA and PSO in benchmark function F12 (Griewank). **c.** Convergence characteristic curves for QLSA, LSA, BSA, GSA and PSO in benchmark function F13 (Penalised) and, **d.** Convergence characteristic curves for QLSA, LSA, BSA, GSA and PSO in benchmark function F14 (Penalised 2)

|  |  |  |
| --- | --- | --- |
| (a) | (b) | (c) |
|  |  |  |
| (d) | (e) | (f) |

**Supplementary Figure 8.** **Simulation results under ramp speed response test. a.** QLSA performance in ramp response test under the speed varying from 105 rad/sec to 140 rad/sec at no-load. **b.** QLSA performance in ramp response test under the speed varying from 105 rad/sec to 140 rad/sec with 2 Nm load. **c.** QLSA performance in ramp response test under the speed varying from 70 rad/sec to 140 rad/sec at no-load. **d.** QLSA performance in ramp response test under the speed varying from 70 rad/sec to 140 rad/sec with 2 Nm load. **e.** QLSA performance in ramp response test under the speed varying from 35 rad/sec to 140 rad/sec at no-load and. **f.** QLSA performance in ramp response test under the speed varying from 35 rad/sec to 140 rad/sec with 1 Nm load.

|  |  |  |
| --- | --- | --- |
| (a) | (b) | (c) |
|  |  |  |
| (d) | (e) | (f) |

**Supplementary Figure 9.** **Experimental results under ramp speed response test. a.** QLSA performance in ramp response test under the speed varying from 105 rad/sec to 140 rad/sec at no-load. **b.** QLSA performance in ramp response test under the speed varying from 105 rad/sec to 140 rad/sec with 2 Nm load. **c.** QLSA performance in ramp response test under the speed varying from 70 rad/sec to 140 rad/sec at no-load. **d.** QLSA performance in ramp response test under the speed varying from 70 rad/sec to 140 rad/sec with 2 Nm load. **e.** QLSA performance in ramp response test under the speed varying from 35 rad/sec to 140 rad/sec at no-load and. **f.** QLSA performance in ramp response test under the speed varying from 35 rad/sec to 140 rad/sec with 1 Nm load.

.

**Supplementary Figure 10.** Speed response for constant load with speed variations in steps.

**Supplementary Figure 11.** Stator currents with the change in the reference speed.

**Supplementary Figure 12.** Full speed response with mechanical load variations.

**Supplementary Figure 13.** Stator currents with change in the mechanical load.

**Supplementary Figure 14.** Block diagram of the closed loop of scalar control for TIM drive


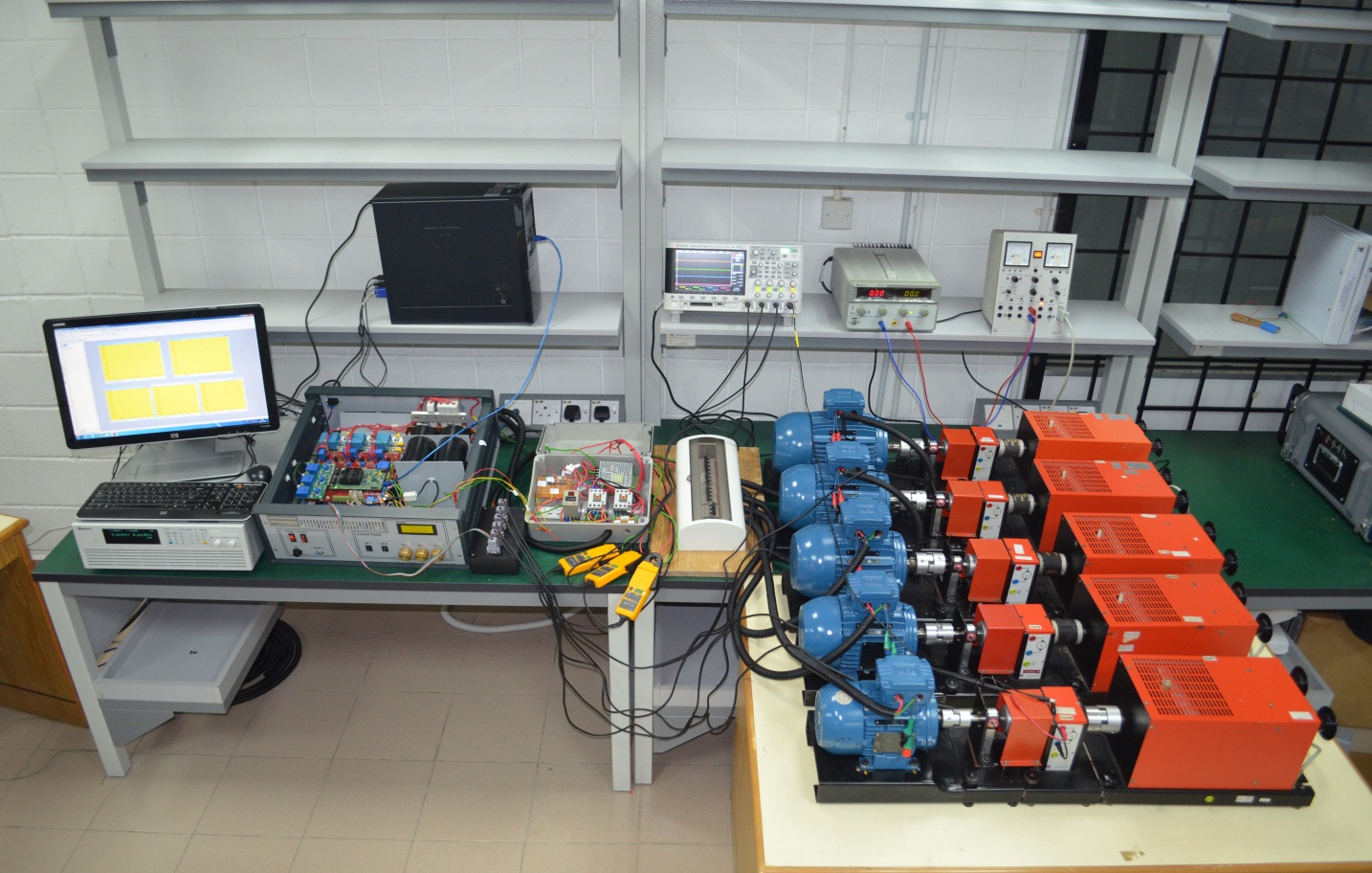


Code Composer Studio (CCS)

Digital Storage Oscilloscope (DSO)

DC Power supply

PC

Magnetic powder brake control unit

Magnetic powder brakes

Tachogenerators

Rotary Encoders

Circuit Breaker

Motor 1 (0.5 HP)

DSP

TMS320F28335

3-ph Inverter

Motor 2 (1 HP)

Motor 3 (1.5 HP)

Motor 4 (2 HP)

Motor 5 (3 HP)

Oscilloscope Current Probes (FLUKE)

Rotary encoder

Multiplexer

Motor selector circuit

DC

Power supply

**Supplementary Figure 15.** Snapshot of the experimental setup of QLSA-based FSC

**Supplementary Tables**

**Supplementary Table 1.** List of 14 benchmark functions for QLSA evaluation

| **Function ID** | **Name** | **Expression** | **Dimension problem, *n*** | **Search space** | **Function minimum** |
| --- | --- | --- | --- | --- | --- |
| F1 | Sphere | $f_{1}\left( x \right)=\sum_{i=1}^{n} x_{i}^{2}$ | 30 | $\left[ -100, 100 \right]^{n}$ | 0 |
| F2 | Step | $f_{2}\left( x \right)=\sum_{i=1}^{n} {(\left\lfloor x_{i}+0.5 \right\rfloor)}^{2}$ | 30 | $\left[ -100, 100 \right]^{n}$ | 0 |
| F3 | Quartic | $f_{3}\left( x \right)=\sum_{i=1}^{n} {ix}_{i}^{4}+rand(0,1)$ | 30 | $\left[ -1.28, 1.28 \right]^{n}$ | 0 |
| F4 | Schwefel 2.22 | $f_{4}\left( x \right)=\sum_{i=1}^{n} \left\vert x_{i} \right\vert+\prod_{i=1}^{n} \left\vert x_{i} \right\vert$ | 30 | $\left[ -10, 10 \right]^{n}$ | 0 |
| F5 | Schwefel 1.2 | $f_{5}\left( x \right)=\sum_{i=1}^{n} \left( \sum_{j=1}^{i} x_{j} \right)^{2}$ | 30 | $\left[ -100, 100 \right]^{n}$ | 0 |
| F6 | Schwefel 2.21 | $f_{6}\left( x \right)={max}_{i} \left\{ \left\vert x_{i} \right\vert, 1\leq i\leq n \right\}$ | 30 | $\left[ -100, 100 \right]^{n}$ | 0 |
| F7 | Rosenbrock | $f_{7}\left( x \right)=\sum_{i=1}^{n-1} \left[ 100\left( x_{i+1}-x_{i}^{2} \right)^{2}+\left( x_{i}-1 \right)^{2} \right]$ | 30 | $\left[ -30, 30 \right]^{n}$ | 0 |
| F8 | Rastrigin | $f_{8}\left( x \right)=\sum_{i=1}^{n} x_{i}^{2}-10\cos\left( 2\pi x_{i} \right)+10$ | 30 | $\left[ -5.12, 5.12 \right]^{n}$ | 0 |
| F9 | Foxholes | $f_{9}\left( x \right)=\left[ \frac{1}{500}+\sum_{j=1}^{25} \frac{1}{j+\sum_{i=1}^{2} \left( x_{i}-a_{ij} \right)^{6}} \right]^{-1}$ | 2 | $\left[ -65.53, 65.53 \right]^{n}$ | 1 |
| F10 | Branin | $f_{10}\left( x \right)=\left( x_{2}-\frac{5.1}{4\pi^{2}}x_{1}^{2}+\frac{5}{\pi}x_{1}-6 \right)^{2}+10\left( 1-\frac{1}{8\pi} \right)cosx_{1}+10$ | 2 | $\left[ -5,10 \right]x[0,15]$ | 0.398 |
| F11 | Ackley | $f_{11}\left( x \right)=-20\exp\left( -0.2\sqrt{\frac{1}{n}\sum_{i=1}^{n} x_{i}^{2}} \right)-\exp\left( \frac{1}{n}\sum_{i=1}^{n} \cos\left( 2\pi x_{i} \right) \right)+20+e$ | 30 | $\left[ -32, 32 \right]^{n}$ | 0 |
| F12 | Griewank | $f_{12}\left( x \right)=\frac{1}{4000}\sum_{i=1}^{n} x_{i}^{2}-\prod_{i=1}^{n} \cos\left( \frac{x_{i}}{\sqrt{i}} \right)+1$ | 30 | $\left[ -600, 600 \right]^{n}$ | 0 |
| F13 | Penalised | $f_{13}\left( x \right)=\frac{\mu}{n}\left\{ 10{sin}^{2}\left( \pi y_{i} \right)+\sum_{i=1}^{n-1} \left( y_{i}-1 \right)^{2}\left[ 1+10{sin}^{2}\left( \pi y_{i+1} \right) \right]+\left( y_{n}-1 \right)^{2} \right\}+\sum_{i=1}^{n} u\left( x_{i},10,100,4 \right)$  $y_{i}=1+\frac{x_{i}+1}{4}$ , $u\left( x_{i},a,k,m \right)=\left\{ \begin{aligned} k{(x_{i}-a)}^{m} \\ 0 \\ k{(-x_{i}-a)}^{m} \end{aligned} \begin{matrix} x_{i}>a \\ -a<x_{i}<a \\ a<x_{i} \end{matrix} \right.$ | 30 | $\left[ -50, 50 \right]^{n}$ | 0 |
| F14 | Penalised 2 | $f_{14}\left( x \right)=0.1\left\{ {sin}^{2}\left( 3\pi x_{1} \right)+\sum_{i=1}^{n-1} \left( x_{i}-1 \right)^{2}\left[ 1+3{sin}^{2}\left( 3\pi x_{i+1} \right) \right]+\left( x_{n}-1 \right)^{2}[1+{sin}^{2}\left( 2\pi x_{n} \right)] \right\}+\sum_{i=1}^{n} u\left( x_{i},5,100,4 \right)$  $u\left( x_{i},a,k,m \right)=\left\{ \begin{aligned} k{(x_{i}-a)}^{m} \\ 0 \\ k{(-x_{i}-a)}^{m} \end{aligned} \begin{matrix} x_{i}>a \\ -a<x_{i}<a \\ a<x_{i} \end{matrix} \right.$ | 30 | $\left[ -50, 50 \right]^{n}$ | 0 |

**Supplementary Table 2.** Fuzzy rules of the induction motor speed controller

$$de$$

$$e$$

|  | Ne3 | Ne2 | Ne1 | Ze | Pe1 | Pe2 | Pe3 |
| --- | --- | --- | --- | --- | --- | --- | --- |
| Nde3 | NB | NB | NB | NB | NM | NS | Z |
| Nde2 | NB | NB | NB | NM | NS | Z | PS |
| Nde1 | NB | NB | NM | NS | Z | PS | PM |
| Zde | NB | NM | NS | Z | PS | PM | PB |
| Pde1 | NM | NS | Z | PS | PM | PB | PB |
| Pde2 | NS | Z | PS | PM | PB | PB | PB |
| Pde3 | Z | PS | PM | PB | PB | PB | PB |

NB: Negative big; NM: Negative medium; NS: Negative small; Z: Zero; PS: Positive small; PM: Positive medium; PB: Positive big.

**Supplementary Table 3.** Unimodal and separable functions test results for benchmark functions (F1 to F3)

| **Function ID** | **Statistics** | **QLSA** | **LSA** | **BSA** | **GSA** | **PSO** |
| --- | --- | --- | --- | --- | --- | --- |
| F1 | Best | **0.000000000** | 7.09870E-19 | 1.120669250 | 1.56570E-17 | 2.23355E-06 |
|  | Worst | **0.000000000** | 8.64142E-10 | 34.94283275 | 8.40797E-17 | 1.61577E-04 |
|  | Median | **0.000000000** | 1.01697E-15 | 6.626990636 | 3.35132E-17 | 2.03621E-05 |
|  | Average | **0.000000000** | 2.60209E-11 | 8.411449411 | 3.52547E-17 | 2.99507E-05 |
|  | Standard Deviation | **0.000000000** | 1.26772E-10 | 6.928749822 | 1.06066E-17 | 3.23395E-05 |
|  |  |  |  |  |  |  |
| F2 | Best | **0.000000000** | 0.000000000 | 1.000000000 | 0.000000000 | 0.000000000 |
|  | Worst | **0.000000000** | 11.00000000 | 42.00000000 | 4.000000000 | 1.000000000 |
|  | Median | **0.000000000** | 3.000000000 | 10.00000000 | 0.000000000 | 0.000000000 |
|  | Average | **0.000000000** | 3.620000000 | 11.54000000 | 0.500000000 | 0.040000000 |
|  | Standard Deviation | **0.000000000** | 2.594263687 | 7.228656125 | 0.952976004 | 0.197948663 |
|  |  |  |  |  |  |  |
| F3 | Best | **6.03786E-07** | 0.036629159 | 0.017554364 | 0.008620920 | 1.060358160 |
|  | Worst | **3.16982E-04** | 0.227804447 | 0.095580649 | 0.077749911 | 119.1095239 |
|  | Median | **4.23113E-05** | 0.087547435 | 0.043287573 | 0.021583480 | 8.284676020 |
|  | Average | **6.03238E-05** | 0.098258947 | 0.048949989 | 0.027740445 | 21.27003034 |
|  | Standard Deviation | **6.36075E-05** | 0.040880054 | 0.017746284 | 0.016309359 | 30.78542892 |

**Supplementary Table 4.** Unimodal and non-separable functions test results for benchmark functions (F4 to F7)

| **Function ID** | **Statistics** | **QLSA** | **LSA** | **BSA** | **GSA** | **PSO** |
| --- | --- | --- | --- | --- | --- | --- |
| F4 | Best | **0.000000000** | 1.82027E-07 | 0.302826963 | 2.08662E-08 | 0.001371894 |
|  | Worst | **0.000000000** | 0.157899025 | 3.520797010 | 4.63805E-08 | 0.018663005 |
|  | Median | **0.000000000** | 7.87338E-04 | 1.017066937 | 3.11027E-08 | 0.003955458 |
|  | Average | **0.000000000** | 0.010597435 | 1.195271023 | 3.16555E-08 | 0.005004374 |
|  | Standard Deviation | **0.000000000** | 0.028400248 | 0.630281571 | 6.06003E-09 | 0.003532853 |
|  |  |  |  |  |  |  |
| F5 | Best | **0.000000000** | 5.478935752 | 1039.968015 | 181.3818591 | 9.227013952 |
|  | Worst | **0.000000000** | 138.8012639 | 4953.536427 | 929.1028885 | 65.52530245 |
|  | Median | **0.000000000** | 38.25290737 | 2657.642073 | 515.8335840 | 27.62900421 |
|  | Average | **0.000000000** | 46.19244109 | 2668.153461 | 526.0800335 | 27.36473672 |
|  | Standard Deviation | **0.000000000** | 30.56948822 | 1035.636549 | 165.0566862 | 11.30292806 |
|  |  |  |  |  |  |  |
| F6 | Best | **0.000000000** | 0.120886090 | 5.596731215 | 0.954938142 | 0.316719290 |
|  | Worst | **0.000000000** | 9.360536140 | 14.13244731 | 7.664633208 | 1.013009797 |
|  | Median | **0.000000000** | 1.210138997 | 9.466250327 | 3.809147629 | 0.563159580 |
|  | Average | **0.000000000** | 2.174150862 | 9.635753831 | 3.973144107 | 0.604233254 |
|  | Standard Deviation | **0.000000000** | 2.287297716 | 2.057804288 | 1.615648420 | 0.154328440 |
|  |  |  |  |  |  |  |
| F7 | Best | 27.57315322 | **1.190409834** | 151.0265579 | 25.08961811 | 7.155048288 |
|  | Worst | **28.95493548** | 162.1883669 | 1576.459154 | 182.1393968 | 152.6767961 |
|  | Median | **28.12805156** | 72.13999562 | 456.7119696 | 29.49619631 | 39.96090973 |
|  | Average | **28.40467584** | 54.50005251 | 521.1076103 | 37.11611644 | 61.38078346 |
|  | Standard Deviation | **0.458110857** | 40.06408143 | 318.5941011 | 28.10767442 | 39.44916900 |

**Supplementary Table 5.** Multimodal and separable functions test results for benchmark functions (F8 to F10)

| **Function ID** | **Statistics** | **QLSA** | **LSA** | **BSA** | **GSA** | **PSO** |
| --- | --- | --- | --- | --- | --- | --- |
| F8 | Best | **0.000000000** | 32.83360349 | 46.34048533 | 6.964713399 | 23.53724665 |
|  | Worst | **0.000000000** | 91.53599093 | 83.40654086 | 30.84370558 | 64.44904395 |
|  | Median | **0.000000000** | 59.69744761 | 62.67504101 | 17.90925295 | 35.31624232 |
|  | Average | **0.000000000** | 61.13016762 | 63.48062046 | 17.45157712 | 38.69440474 |
|  | Standard Deviation | **0.000000000** | 13.67934961 | 8.491075435 | 4.544869878 | 9.512522510 |
|  |  |  |  |  |  |  |
| F9 | Best | **0.998003837** | **0.998003837** | **0.998003837** | **0.998003837** | **0.998003837** |
|  | Worst | 2.982105159 | 3.968250105 | **0.998003837** | 12.67247904 | 5.928845125 |
|  | Median | 0.998003840 | 0.998003837 | **0.998003837** | 4.660449301 | 1.992030900 |
|  | Average | 1.831326393 | 1.116813688 | **0.998003837** | 5.310301946 | 1.791179860 |
|  | Standard Deviation | 0.989212167 | 0.587956279 | **2.03111E-16** | 3.160586200 | 1.093134459 |
|  |  |  |  |  |  |  |
| F10 | Best | **0.397890111** | 0.397887357 | 0.397887357 | 0.397887357 | 0.397887357 |
|  | Worst | 0.400181760 | **0.397887357** | **0.397887357** | **0.397887357** | **0.397887357** |
|  | Median | **0.398182078** | 0.397887357 | 0.397887357 | 0.397887357 | 0.397887357 |
|  | Average | 0.398366924 | **0.397887357** | **0.397887357** | **0.397887357** | **0.397887357** |
|  | Standard Deviation | 4.94181E-04 | **3.36448E-16** | 3.35564E-13 | **3.36448E-16** | **3.36448E-16** |

**Supplementary Table 6.** Multimodal and non-separable functions test results for benchmark functions (F11 to F14)

| **Function ID** | **Statistics** | **QLSA** | **LSA** | **BSA** | **GSA** | **PSO** |
| --- | --- | --- | --- | --- | --- | --- |
| F11 | Best | **8.88178E-16** | 3.64345E-09 | 0.995397591 | 3.45695E-09 | 8.90922E-04 |
|  | Worst | **8.88178E-16** | 5.319336794 | 8.375948902 | 6.68617E-09 | 0.043437712 |
|  | Median | **8.88178E-16** | 2.316161813 | 2.922905313 | 4.81967E-09 | 0.003053435 |
|  | Average | **8.88178E-16** | 2.465444740 | 3.198420835 | 4.77382E-09 | 0.003927735 |
|  | Standard Deviation | **0.000000000** | 1.121549616 | 1.735282315 | 6.09597E-10 | 0.005874239 |
|  |  |  |  |  |  |  |
| F12 | Best | **0.000000000** | 1.11022E-16 | 0.904806697 | 11.65911615 | 1.62825E-07 |
|  | Worst | **0.000000000** | 0.031942367 | 1.365261133 | 25.95643987 | 0.078655565 |
|  | Median | **0.000000000** | 0.007396040 | 1.096906421 | 16.82627141 | 2.99897E-05 |
|  | Average | **0.000000000** | 0.008566486 | 1.116238499 | 17.42329001 | 0.011796166 |
|  | Standard Deviation | **0.000000000** | 0.009219876 | 0.082051221 | 3.415560583 | 0.018155994 |
|  |  |  |  |  |  |  |
| F13 | Best | 0.004754723 | **1.41869E-18** | 0.009842130 | 3.22078E-05 | 1.32277E-08 |
|  | Worst | **0.020247708** | 3.550460150 | 1.172647377 | 2.119972106 | 0.103669944 |
|  | Median | 0.009868975 | 0.103669020 | 0.090089702 | 0.502477716 | **1.99187E-07** |
|  | Average | **0.009928269** | 0.378148584 | 0.151495738 | 0.633387890 | 0.012440681 |
|  | Standard Deviation | **0.002535644** | 0.684871874 | 0.195685896 | 0.513519531 | 0.034030477 |
|  |  |  |  |  |  |  |
| F14 | Best | 3.16120E-04 | **3.07485E-04** | 4.41061E-04 | 0.001182912 | **3.07487E-04** |
|  | Worst | **7.62676E-04** | 0.001594049 | 8.13040E-04 | 0.012475146 | 0.020363339 |
|  | Median | 4.23875E-04 | **3.16631E-04** | 7.06902E-04 | 0.004030443 | 3.60942E-04 |
|  | Average | **4.37709E-04** | 5.52853E-04 | 6.78746E-04 | 0.004242917 | 8.92626E-04 |
|  | Standard Deviation | **9.71587E-05** | 4.21531E-04 | 1.04481E-04 | 0.001992115 | 0.002822596 |

**Supplementary Table 7.** Step response test simulation results under different speed responses

| Mechanical load | | | No-load | | | 2 Nm | | |
| --- | --- | --- | --- | --- | --- | --- | --- | --- |
| Speed (rad/sec) | Controller type | | MAE | RMSE | SD | MAE | RMSE | SD |
| 105 to 140 | QLSAF | | **3.1761** | **16.4127** | **16.1567** | **3.4720** | **16.5700** | **16.2653** |
|  | LSAF | | 3.1822 | 16.4134 | 16.1635 | 3.4999 | 16.5844 | 16.2751 |
|  | BSAF | | 3.2038 | 16.4204 | 16.1816 | 3.5741 | 16.6577 | 16.3346 |
|  | GSAF | | 3.2188 | 16.4207 | 16.1789 | 3.6505 | 16.6854 | 16.3353 |
|  | PSOF | | 3.2190 | 16.4284 | 16.1872 | 3.6819 | 16.7417 | 16.3888 |
|  |  | | | | | 2 Nm | | |
| 70 to 140 | QLSAF | | **1.9064** | **10.2400** | **10.1116** | **2.1009** | **10.4828** | **10.2860** |
|  | LSAF | | 1.9094 | 10.2497 | 10.1142 | 2.1098 | 10.4871 | 10.3087 |
|  | BSAF | | 1.9502 | 10.2726 | 10.1375 | 2.1146 | 10.4928 | 10.3116 |
|  | GSAF | | 2.0199 | 10.3623 | 10.2468 | 2.1157 | 10.5173 | 10.3243 |
|  | PSOF | | 2.2589 | 10.6997 | 10.6004 | 2.1302 | 10.5198 | 10.3388 |
|  |  | | | | | 1 Nm | | |
| 35 to 140 | QLSAF | **1.4842** | | **5.9988** | **5.9608** | **1.6872** | **6.5379** | **6.4662** |
|  | LSAF | 1.6185 | | 6.4238 | 6.4035 | 1.6942 | 6.6376 | 6.5895 |
|  | BSAF | 1.7380 | | 6.8825 | 6.8647 | 1.7111 | 6.7405 | 6.6933 |
|  | GSAF | 1.8784 | | 7.4470 | 7.4344 | 1.7756 | 6.9452 | 6.8997 |
|  | PSOF | 1.9854 | | 7.6817 | 7.6630 | 1.8289 | 7.0009 | 6.9429 |

**Supplementary Table 8.** The DTUTD Speed Response test simulation results under different loads

| Load (Nm) | Controller type | MAE | RMSE | SD |
| --- | --- | --- | --- | --- |
| No-load | QLSAF | **0.7493** | **4.5101** | **4.4964** |
|  | LSAF | 0.7495 | 4.5108 | 4.4985 |
|  | BSAF | 0.7531 | 4.5258 | 4.5156 |
|  | GSAF | 0.7723 | 4.5316 | 4.5170 |
|  | PSOF | 0.7834 | 4.5677 | 4.5521 |
|  |  |  |  |  |
| 1 Nm | QLSAF | **0.7977** | **4.6566** | **4.6430** |
|  | LSAF | 0.8100 | 4.6618 | 4.6501 |
|  | BSAF | 0.8141 | 4.6729 | 4.6583 |
|  | GSAF | 0.8141 | 4.6912 | 4.6749 |
|  | PSOF | 0.8606 | 4.7506 | 4.7325 |
|  |  | | | |
| 2 Nm | QLSAF | **1.1879** | **7.7192** | **7.6507** |
|  | LSAF | 1.1995 | 7.7301 | 7.6623 |
|  | BSAF | 1.2182 | 7.7072 | 7.6683 |
|  | GSAF | 1.2733 | 7.7814 | 7.7146 |
|  | PSOF | 1.2733 | 7.7814 | 7.7146 |

**Supplementary Table 9.** The ramp response test result under different speed responses

| Mechanical load | | | No-load | | | 2 Nm | | |
| --- | --- | --- | --- | --- | --- | --- | --- | --- |
| Speed (rad/sec) | Controller type | | MAE | RMSE | SD | MAE | RMSE | SD |
| 105 to 140 | QLSAF | | **2.9980** | **16.6553** | **16.3869** | **3.5827** | **17.4315** | **17.0585** |
|  | LSAF | | 3.0022 | 16.6561 | 16.3899 | 3.5885 | 17.4321 | 17.0603 |
|  | BSAF | | 3.0193 | 16.6603 | 16.3986 | 3.5906 | 17.4321 | 17.0616 |
|  | GSAF | | 3.0198 | 16.6606 | 16.3986 | 3.5956 | 17.4321 | 17.0629 |
|  | PSOF | | 3.0349 | 16.6610 | 16.4011 | 3.5978 | 17.4323 | 17.0633 |
|  | No-load | | | | | 2 Nm | | |
| 70 to 140 | QLSAF | | **1.8834** | **10.9473** | **10.7862** | **2.0831** | **11.1266** | **10.9286** |
|  | LSAF | | 1.8849 | 10.9474 | 10.7879 | 2.0862 | 11.1266 | 10.9292 |
|  | BSAF | | 1.8989 | 10.9518 | 10.7957 | 2.0931 | 11.1266 | 10.9301 |
|  | GSAF | | 1.9007 | 10.9521 | 10.7962 | 2.0971 | 11.1286 | 10.9307 |
|  | PSOF | | 1.9158 | 10.9521 | 10.7987 | 2.1002 | 11.1302 | 10.9312 |
|  | No-load | | | | | 1 Nm | | |
| 35 to 140 | QLSAF | **0.8874** | | **5.5107** | **5.4409** | **0.8943** | **5.5129** | **5.4436** |
|  | LSAF | 0.8927 | | 5.5124 | 5.4422 | 0.8975 | 5.5130 | 5.4438 |
|  | BSAF | 0.8967 | | 5.5147 | 5.4467 | 0.9041 | 5.5156 | 5.4442 |
|  | GSAF | 0.8985 | | 5.5147 | 5.4475 | 0.9063 | 5.5172 | 5.4444 |
|  | PSOF | 0.9128 | | 5.5149 | 5.4498 | 0.9139 | 5.5195 | 5.4446 |

**Supplementary Table 10.** The speed response results of QLSAF, LSAF, BSAF, GSAF, PSOF, and PID controllers against the changes in the reference speed.

| **Period (s)** | **Reference speed (rad/s)** | **Maximum overshoot (%)** | | | | | |
| --- | --- | --- | --- | --- | --- | --- | --- |
|  |  | **QLSAF** | **LSAF** | **BSAF** | **GSAF** | **PSOF** | **PID control** |
| 0–0.5 | 157 | 4.45 | 7.64 | 7.00 | 12.10 | 12.11 | 13.15 |
| 0.5–0.7 | 118 | 1.27 | 5.08 | 8.47 | 2.96 | 9.32 | 9.81 |
| 0.7–0.9 | 78 | 0.64 | 4.48 | 7.69 | 0.71 | 10.21 | 10.92 |
| 0.9–1.4 | 39 | 1.28 | 7.69 | 10.2 | 1.32 | 12.82 | 16.67 |
| 1.4–1.6 | 78 | 2.56 | 3.84 | 3.20 | 10.26 | 6.41 | 12.30 |
| 1.6–1.8 | 118 | 2.11 | 2.54 | 2.33 | 6.77 | 6.81 | 8.305 |
| 1.8–2 | 157 | 2.54 | 2.86 | 2.54 | 7.00 | 7.21 | 8.280 |
| **Period (s)** | **Reference speed (rad/s)** | **Settling time (s)** | | | | | |
|  |  | **QLSAF** | **LSAF** | **BSAF** | **GSAF** | **PSOF** | **PID Control** |
| 0–0.5 | 157 | 0.054 | 0.055 | 0.055 | 0.06 | 0.07 | 0.125 |
| 0.5–0.7 | 118 | 0.011 | 0.014 | 0.015 | 0.012 | 0.025 | 0.042 |
| 0.7–0.9 | 78 | 0.009 | 0.012 | 0.018 | 0.011 | 0.026 | 0.055 |
| 0.9–1.4 | 39 | 0.009 | 0.010 | 0.015 | 0.011 | 0.024 | 0.125 |
| 1.4–1.6 | 78 | 0.015 | 0.010 | 0.010 | 0.011 | 0.027 | 0.056 |
| 1.6–1.8 | 118 | 0.018 | 0.014 | 0.011 | 0.012 | 0.022 | 0.041 |
| 1.8–2 | 157 | 0.015 | 0.016 | 0.012 | 0.017 | 0.027 | 0.044 |

**Supplementary Table 11.** Detailed speed response results of QLSAF, LSAF, BSAF, GSAF, PSOF, and PID controllers against the changes in mechanical load

| **Period (s)** | **Load (N-m)** | **Maximum overshoot (%)** | | | | | | **Settling time (s)** | | | | | | |
| --- | --- | --- | --- | --- | --- | --- | --- | --- | --- | --- | --- | --- | --- | --- |
|  |  | **QLSAF** | **LSAF** | **BSAF** | **GSAF** | **PSOF** | **PID control** | **QLSAF** | **LSAF** | **BSAF** | **GSAF** | **PSOF** | **PID control** |  |
| 0–0.3 | 0 | **4.45** | 7.64 | 7.00 | 12.10 | 12.11 | 13.15 | **0.0540** | 0.0550 | 0.0550 | 0.0600 | 0.0700 | 0.125 |  |
| 0.3–0.5 | 1.247 | **0.127** | 0.159 | 0.191 | 0.382 | 0.254 | 1.464 | **0.0030** | 0.0032 | 0.0031 | 0.0035 | 0.0200 | 0.011 |  |
| 0.5–0.7 | 2.495 | **0.127** | 0.191 | 0.222 | 0.350 | 0.254 | 1.745 | **0.0032** | **0.0032** | **0.0032** | 0.003 | 0.0160 | 0.032 |  |
| 0.7–0.9 | 3.742 | **0.095** | 0.159 | 0.159 | 0.318 | 0.223 | 2.292 | **0.0045** | **0.0045** | **0.0045** | 0.0055 | 0.0200 | 0.063 |  |
| 0.9–1.2 | 4.990 | **0.159** | 0.178 | 0.192 | 0.286 | 0.224 | 2.484 | **0.0050** | **0.0050** | **0.0050** | **0.0050** | 0.0180 | 0.035 |  |
| 1.2–1.4 | 3.742 | **0.445** | 0.477 | 0.445 | 0.507 | 0.477 | 1.401 | **0.0020** | 0.0025 | 0.0025 | 0.0030 | 0.0200 | 0.042 |  |
| 1.4–1.6 | 2.495 | **0.371** | 0.371 | 0.445 | 0.477 | 0.445 | 1.783 | **0.0020** | 0.0045 | 0.0045 | 0.0025 | 0.0200 | 0.022 |  |
| 1.6–1.8 | 1.247 | **0.382** | 0.392 | 0.426 | 0.509 | 0.445 | 2.038 | **0.0020** | 0.0055 | 0.0060 | 0.006 | 0.0150 | 0.023 |  |
| 1.8–2 | 0 | 0.392 | **0.382** | 0.477 | 0.509 | 0.482 | 2.292 | **0.0020** | 0.0050 | 0.0060 | 0.005 | 0.0200 | 0.021 |  |

**Supplementary Notes**

**Supplementary Note 1: Lightning Search Algorithm**

LSA optimisation technique is based on the mechanism of step leader propagation of lightning^1^. It considers the involvement of fast particles known as projectiles in the formation of the binary tree structure of the step leader and in the concurrent formation of two leader tips at fork points instead of a conventional step leader mechanism. LSA mechanism consists of three steps; projectile and step leader propagation, projectile properties, and projectile modelling and movement.

**Projectile and Step Leader Propagation*.*** There are many atoms near the thunderclouds such as hydrogen, nitrogen, oxygen and also the intensive freezing of water molecules by forming ice at intense speeds which lead to separate hydrogen and oxygen atoms and ejected in random direction as projectiles. LSA technique considers each projectile representing the initial population size.

**Projectile Properties.** The projectile is moving in the atmosphere but loses its kinetic energy during elastic collisions with molecules and atoms in the air. The projectile velocity is given by,

$$v_{p}=\left[ 1-\left( \frac{1}{\sqrt{1-\left( {v_{0}}/c \right)^{2}}}-\frac{sF_{i}}{mc^{2}} \right)^{-2} \right]^{{-1}/2} \left( 1 \right)$$

where $v_{p}$ is current velocity of the projectile; $v_{0}$ initial velocity of the projectile; $F_{i}$ is the constant ionization rate $c$ is the light speed; m is the mass of the projectile; and $s$ is the length of the path travelled.

The number of projectiles is increased through add channels created during forking which leads to increase population size. LSA technique creates forking by two methods. The first method is process generate symmetrical channels because the nuclei collision of the projectile is realised by using the opposite number as show below.

$\bar{p}_{i}=a+b-p_{i} (2)$

where$\bar{p}_{i}$ is opposite projectiles in one-dimension, $a$ and $b$ are the boundary limits, and $p_{i}$ is original projectiles in one-dimension.

The population may improve in some complex solution. In the second type of forking, a channel is assumed to appear at a successful step leader tip because of the energy redistribution of the most unsuccessful leader after several propagation trials. The unsuccessful leader can be redistributed by defining the maximum allowable number of trials as channel time. In this case, the population size of step leaders does not increase.

**Projectile Modelling and Movement.** There are three types of projectiles to represent the transition projectiles that generate the first-step leader population *N*. Transition projectile may be random direction through the transition forms an ejected projectile from the thunder cell. Therefore, transition projectile can represent a random number through create form of the random distribution in the space. The probability density function $f\left( x^{T} \right)$ of the standard uniform distribution can be represented in equation below.

$$f\left( x^{T} \right)=\left\{ {1/(b-a) \atop0} \right. \begin{matrix} for \\ for \end{matrix} \begin{matrix} a\leq x^{T}\leq b \\ x<a or x^{T}>b \end{matrix} (3)$$

where $x^{T}$ is a random values to represent the initial tip energy, $E_{sl\_i}$ of the step leader, ${sl}_{i}$; $a$ is lower bound of the solution space; $b$ is upper bound of the solution space; $SL=\left[ {sl}_{1}, {sl}_{2}, {sl}_{3},\ldots\ldots, {sl}_{N} \right]$ are step leaders for population of *N*, and $P^{T}=\left[ P_{1}^{T},P_{2}^{T},P_{3}^{T},\ldots\ldots, P_{N}^{T} \right]$ are the solution dimension are required for each population. The leaders population move to space depend on activity projectiles and ionizing the section in the vicinity of the old leader tip in the next step. The new position of projectile is distributed by form called exponential distribution with shaping parameter$\mu$. The probability densities function$f\left( x^{s} \right)$of an exponential distribution is given by,

$$f\left( x^{s} \right)=\left\{ \begin{matrix} \frac{1}{\mu}e^{-\frac{x^{s}}{\mu}} & for & x^{s}\geq0 \\ 0 & for & x^{s}\leq0 \end{matrix} \right. (4)$$

where$\mu_{i}$ is shaping parameter. In the LSA mechanism, $\mu_{i}$ is the distance between the lead projectile, $p^{L}$ and the space projectile, $p_{i}^{S}$ under consideration. The new position is defined as,

$$p_{i_{new}}^{s}=p_{i}^{s}\pm exprand\left( \mu_{i} \right) (5)$$

where $\mathrm{exprand}$ is an exponential random number for $\mu_{i}$. The new position, $p_{i_{new}}^{s}$ may find a good solution obtaining new position, $p_{i_{new}}^{s}$ and update to$p_{i}^{s}$. Otherwise, they remain in unchanged position until the next step. The step leaders when access nearest to ground and the projectile associated with it do not have enough potential to ionize large sections in front of the leader tip. Therefore, the lead projectile can be represented as a random number generated by the standard normal distribution with the shape parameter $\mu$and the scale parameter$\sigma$. The normal probability density function$f(x^{L})$ is expressed as,

$$f\left( x^{L} \right)=\frac{1}{\sigma\sqrt{2\pi}}e^{-\frac{\left( x^{L}-\mu\right)^{2}}{2\sigma^{2}}} (6)$$

In LSA technique, $\sigma_{L}$ is a scalar parameter exponentially decreasing as it finds the best solution, $\mu_{L}$for the lead projectile,$p^{L}$. The new position of $p^{L}$ is represented by,

$$p_{new}^{L}=p^{L}+normrand\left( \mu_{L},\sigma_{L} \right) (7)$$

where $normrand$ is a random number generated by the normal distribution function. The flowchart of the optimal fuzzy speed controller using LSA is shown in supplementary Fig. 2.

**Supplementary Note 2: Backtracking Search Algorithm**

BSA optimisation technique is an evolutionary computation technique for producing a trial population which includes two new crossovers and mutation operators^2^. BSA dominates the value of the search on the best populations and in the spaces boundary to find the very sturdy exploration and exploitation capabilities. Thus, it has been proven in considerable research as one of the most powerful optimisation techniques. BSA structure consists of five parts: initialization, selection-I, mutation, crossover, and selection-II. Initialisation process is the primitive configuration of population for the numerical values of population demonstrated by the following equation

$$X_{ij}=rand.\left( {up}_{j}-{low}_{j} \right)+{low}_{j} (8)$$

where $i=1,2,\ldots\ldots,N$, $N$ is population size, $j=1,2,\ldots\ldots,D$, $D$ is the problem dimension. The historical population (${oldX}_{ij}$) to be used for calculating the search direction is constructed by using the following equation:

$${oldX}_{ij}=rand.\left( {up}_{j}-{low}_{j} \right)+{low}_{j} (9)$$

The${oldX}_{ij}$remembers the population from a randomly chosen previous generation for creating the search-direction matrix, taking partial advantage of previous experiences to generate a new trial population. Comparison between two random values is shown in the following condition:

$$if a<b then \mathrm{oldX}_{\mathrm{ij}}:=X_{\mathrm{ij}} \left( 10 \right)$$

$$\mathrm{oldX}_{\mathrm{ij}}=permuting\left( \mathrm{oldX}_{\mathrm{ij}} \right) (11)$$

Mutation is a process that produces the new population of the initial and history population, as shown in Supplementary Equation (11), in which *F* value controls the amplitude of the search-direction matrix.

$$Mutant=X_{ij}+F.randn.\left( oldX_{ij}-X_{ij} \right) (12)$$

BSA generates a trial population, and then takes a partial advantage of its experiences from previous generations. Crossover is generated of the trial population. The initial of the trial populations is taken from mutation, as shown in the Supplementary Equation (12). The crossover consists of two parts. The first part generates the binary matrix called${map}_{ij}$, and the second part is process comparison between population $X_{ij}$ and trial population. Crossover is used to obtain updates${map}_{ij}$. In addition, this part works on control mechanism of boundaries for the trial population. The last part is selection-II. In this part, optimisation process runs to compare the population $X_{ij}$ and trial population to obtain the best population as well as objective value.

**Supplementary Note 3: Gravitational Search Algorithm**

GSA is classified as physics based algorithm it depending on the law of gravity and mass interactions^3^. The operating principle of GSA is based on the laws of motion and the law of Newtonian gravity, which states that ‟every particle in the universe attracts every other particle with a force that is directly proportional to their masses and inversely proportional to the square of the distance between them” as shown in the equation below,

$$F=G \frac{M_{1}M_{2}}{R^{2}} \left( 13 \right)$$

where $F$ is the magnitude of the gravitational force; $G$ is the gravitational constant; $M_{1}$ and $M_{2}$ are the mass of the first and second particles, respectively; and $R$ is the distance between the two particles. According to Newton’s second law, acceleration $a$ is directly proportional to force and inversely proportional to mass $M$ as follows:

$$a= \frac{F}{M} \left( 14 \right)$$

Gravitational constant $G\left( t \right)$ is calculated as the initial value of the gravitational constant, $G\left( t_{0} \right)$, multiplied by the ratio between initial time $t_{0}$ and actual time $t$ as follows:

$$G\left( t \right)=G\left( t_{0} \right)\times\left( \frac{t_{0}}{t} \right)^{\beta}, \beta<1 \left( 15 \right)$$

The positions of the *N* number of the initialisation agents are initialised (i.e., the masses are randomly selected within the given search interval) shown as follows:

$$X_{i}=\left( X_{i}^{1},\ldots\ldots,X_{i}^{d},\ldots\ldots,X_{i}^{n} \right), for i=1,2,\ldots,N \left( 16 \right)$$

where $X_{i}^{d}$ is the position of *i-th* agent in the d-th dimension and n is the space dimension. The computation, which aims to minimise problems and determine the masses of each agent, is as follows:

$$best\left( t \right)=\min_{j\in\left\{ 1,\ldots,N \right\}} {fit}_{j}\left( t \right) (17)$$

$$Worst\left( t \right)=\max_{j\in\left\{ 1,\ldots,N \right\}} {fit}_{j}\left( t \right) (18)$$

$$m_{i}\left( t \right)=\frac{{fit}_{i}\left( t \right)-Worst\left( t \right)}{best\left( t \right)-Worst\left( t \right)} (19)$$

$$M_{i}\left( t \right)=\frac{m_{i}\left( t \right)}{\sum_{j=1}^{N} m_{i}\left( t \right)} \left( 20 \right)$$

The total force *F* computation in different directions in the *i-th* agent, the acceleration a, velocity computation *V*, the position *X* and gravitational constant $G$ of the agents at the next iteration t are as follows:

$$G\left( t \right)=G_{0}e^{\left( -{\alpha t}/T \right)} (21)$$

$$F_{ij}^{d}\left( t \right)=G\left( t \right)\frac{M_{pi}\times M_{aj}}{R_{ij}+\varepsilon}\left( X_{j}^{d}\left( t \right)-X_{i}^{d}\left( t \right) \right) \left( 22 \right)$$

$$F_{i}^{d}\left( t \right)=\sum_{j\in Kbest,j\neq i} {rand}_{j}F_{ij}^{d}\left( t \right) (23)$$

$$a_{i}^{d}\left( t \right)=\frac{F_{i}^{d}\left( t \right)}{M_{i}\left( t \right)} \left( 24 \right)$$

$$v_{i}^{d}\left( t+1 \right)={rand}_{i}\times v_{i}^{d}\left( t \right)+a_{i}^{d}\left( t \right) (25)$$

$$x_{i}^{d}\left( t+1 \right)=x_{i}^{d}\left( t \right)+v_{i}^{d}\left( t+1 \right) \left( 26 \right)$$

**Supplementary Note 4:** **Particle Swarm Optimisation**

PSO is an evolutionary computation technique which is inspired by the social behaviour of bird flocking^4^. The particles in the PSO algorithm search the space in two locations. The first location is the best point where the swarm finds the current iteration (local best). The second location is the best point found through all previous iterations (global best). The principle of the PSO algorithm depends on two factors, namely, velocity and position of particles. These factors can be updated by using the following equations,

$$V_{i}^{d}\left( t+1 \right)={wV}_{i}^{d}\left( t \right)+c_{1}r_{1}\left( P_{i}^{d}\left( t \right)-X_{i}^{d}\left( t \right) \right)+c_{2}r_{2}\left( P_{t}^{d}\left( t \right)-X_{i}^{d}\left( t \right) \right) \left( 27 \right)$$

$${X_{i}^{d}\left( t+1 \right)=X_{i}^{d}\left( t \right)+V}_{i}^{d}\left( t+1 \right) \left( 28 \right)$$

where$c_{1}$ is the social rate, and$c_{2}$ is the cognitive rate. $r_{1}\mathrm{and}r_{2}$denote the random in the interval (0,1). $V$ is the velocity factor of agent $i$ at iteration $d$, $t$ is the present iteration, $w$ is the inertia factor, and $X$ is the position factor.

**Supplementary Note 5: Comparison between QLSA and PID controllers**

**Constant Torque with Speed Variation.** The first test involves increasing or decreasing the reference speed while maintaining a fixed torque. This case study aims to evaluate the performance of the proposed FLC and to estimate the reference speed variation with the constant torque of the TIM controlled by the V/F ratio. V/F control generally exhibits weak performance in low-speed applications. However, V/F ratio controls 25%–100% of the nominal speed of the TIM. The performance of the developed FLC in terms of reference speed involves step responses. The performance of the TIM drive during step response change is determined under the condition of a constant torque load applied on the TIM rotor shaft. By contrast, the no-load condition is applied on the TIM with variable speed in short periods, as illustrated in supplementary Fig. 10. A controller is used to sustain TIM performance. This study proposes a unique robust controller structure to indicate the speed responses of QLSAF, LSAF, BSAF, GSAF, PSOF and PID with a nearly perfect speed change. Supplementary Table 10 shows the speed response, which varies based on specific durations, and the overshoot (%) values. The maximum overshoot is calculated as$maximum overshoot \left( \% \right)=(\frac{N_{\mathrm{overshoot}}-N_{\mathrm{rated}}}{N_{\mathrm{rated}}})\times100\%$. QLSAF successfully achieves the best result compared to the other optimization algorithms in terms of maximum overshoot values and settling time. QLSAF achieves better responses than that of LSAF, BSAF, GSAF, PSOF and PID in terms of minimizing overshoot values, settling time, steady-state error and the damping ratio. After each change, QLSAF establishes excellent rapid stability during each speed change. None of these results can be obtained without a perfect controller, such as the one proposed in this study. Supplementary Figure 11 shows that the stator current signal during the start-up of the TIM involves a high current pull and, subsequently, stable signals. The changes in frequency of the peak value are also fixed during the duration of sudden changes in speed based on system requirements. Controlling speed change corresponds to a change in supply frequency.

**Constant Speed with Torque Variation****.** This test aims to determine system performance and robustness of the proposed controller at full rotor speed with changes in mechanical loads, as illustrated in supplementary Fig. 12. The speed response and its zoomed locations for each step when load changes are shown in supplementary Fig. 12. The estimated speed is considered consistent with the actual speed with good accuracy. In terms of the steady-state error between the reference and actual speeds and damping minimization, QLSAF obtains a better response than LSAF, BSAF, GSAF, PSOF and PID. supplementary Figure 13 presents the stator currents where the constant frequency and variable peak values are modified by step changes in mechanical load for a specific duration. Supplementary Table 11 lists the mechanical load variations according to specific time durations, settling time and overshoot (%) values. QLSAF achieves the lowest overshoot values among the controllers, and these values allow for inducing the best response.

**Supplementary References**

1. Shareef, H., Ibrahim, A. A. & Mutlag, A. H. Lightning search algorithm. *Appl. Soft Comput.* **36**, 315–333 (2015).

2. Ali, J. A., Hannan, M. A., Mohamed, A. & Abdolrasol, M. G. M. Fuzzy logic speed controller optimization approach for induction motor drive using backtracking search algorithm. *Measurement* **78**, 49–62 (2016).

3. Grewal, G. S. & Singh, B. Efficiency determination of in-service induction machines using gravitational search optimization. *Measurement* **118**, 156–163 (2018).

4. Sudha Letha, S., Thakur, T. & Kumar, J. Harmonic elimination of a photo-voltaic based cascaded H-bridge multilevel inverter using PSO (particle swarm optimization) for induction motor drive. *Energy* **107**, 335–346 (2016).
